# Inhibition of *miR‐21* alleviated cardiac perivascular fibrosis via repressing EndMT in T1DM

**DOI:** 10.1111/jcmm.14800

**Published:** 2019-11-03

**Authors:** Qianqian Li, Yufeng Yao, Shumei Shi, Mengchen Zhou, Yingchao Zhou, Mengru Wang, Jeng‐Jiann Chiu, Zhengrong Huang, Weili Zhang, Min Liu, Qing Wang, Xin Tu

**Affiliations:** ^1^ Key Laboratory of Molecular Biophysics of the Ministry of Education College of Life Science and Technology and Center for Human Genome Research Huazhong University of Science and Technology Wuhan China; ^2^ Institute of Cellular and System Medicine National Health Research Institutes Miaoli Taiwan; ^3^ Department of Cardiology The First Affiliated Hospital of Xiamen University Xiamen China; ^4^ State Key Laboratory of Cardiovascular Disease Hypertension Center FuWai Hospital National Center for Cardiovascular Diseases Chinese Academy of Medical Sciences (CAMS) & Peking Union Medical College (PUMC) Beijing China; ^5^ Hypertension Department of Henan Provincial People's Hospital Henan China; ^6^ Center for Cardiovascular Genetics Department of Molecular Cardiology Cleveland Clinic Cleveland OH USA; ^7^ Department of Genetics and Genome Sciences Case Western Reserve University Cleveland OH USA

**Keywords:** cardiac perivascular fibrosis, diabetic cardiomyopathy, endothelial‐mesenchymal transition, microRNA, SMAD7

## Abstract

In type 1 and type 2 diabetes mellitus, increased cardiac fibrosis, stiffness and associated diastolic dysfunction may be the earliest pathological phenomena in diabetic cardiomyopathy. Endothelial‐mesenchymal transition (EndMT) in endothelia cells (ECs) is a critical cellular phenomenon that increases cardiac fibroblasts (CFs) and cardiac fibrosis in diabetic hearts. The purpose of this paper is to explore the molecular mechanism of *miR‐21* regulating EndMT and cardiac perivascular fibrosis in diabetic cardiomyopathy. In vivo, hyperglycaemia up‐regulated the mRNA level of *miR‐21*, aggravated cardiac dysfunction and collagen deposition. The condition was recovered by inhibition of *miR‐21* following with improving cardiac function and decreasing collagen deposition. *miR‐21* inhibition decreased cardiac perivascular fibrosis by suppressing EndMT and up‐regulating SMAD7 whereas activating p‐SMAD2 and p‐SMAD3. In vitro, high glucose (HG) up‐regulated *miR‐21* and induced EndMT in ECs, which was decreased by inhibition of *miR‐21*. A highly conserved binding site of NF‐κB located in *miR‐21* 5′‐UTR was identified. In ECs, SMAD7 is directly regulated by *miR‐21*. In conclusion, the pathway of NF‐κB/*miR‐21*/SMAD7 regulated the process of EndMT in T1DM, in diabetic cardiomyopathy, which may be regarded as a potential clinical therapeutic target for cardiac perivascular fibrosis.

## 1 INTRODUCTION

Type 1 and type 2 diabetes mellitus (T1DM and T2DM) increased the risk of heart failure (HF),^1^, ^2^ thereby inducing cardiomyopathy (referred to as diabetic cardiomyopathy), despite of the other cardiac risk factors, for instance, hypertension, atherosclerosis and valvular disease.^3^, ^4^, ^5^

Cardiac fibrosis and early‐stage left ventricular (LV) hypertrophy are the characteristics of diabetic cardiomyopathy,^3^, ^4^ which often progresses to heart failure with reduced ejection fraction (HFrEF).^6^ In T1DM and T2DM, increased cardiac fibrosis, stiffness and associated diastolic dysfunction possibly are the earliest pathological varieties of diabetic cardiomyopathy.^7^ The underlying pathological mechanisms including perivascular and cardiac interstitial fibrosis and myocardial cell death in cardiac fibrosis pathogenesis in diabetic cardiomyopathy are well acknowledged,^8^ but most studies evaluated T2DM patients and animal models. However, the effect of T1DM on cardiac fibrosis is still unclear.^9^

The protein expression of myocardial extracellular matrix (ECM), especially collagen type I and III, is often elevated in T1DM and T2DM.^10^, ^11^ Excessive production and accumulation of ECM proteins 4 and an out‐off‐balance between MMPs and TIMPs is considered to be one of the main reasons of cardiac fibrosis in diabetic patients.^12^ Cardiac fibroblasts (CFs) are the main origin of ECM proteins, and excessive deposition of ECM proteins is always accompanied by abnormal proliferation of CFs in cardiac fibrosis pathogenesis and progression.^13^

Endothelial‐to‐mesenchymal transition (EndMT) in endothelia cells (ECs) is an important cellular phenomenon that increases CFs and cardiac fibrosis in diabetic hearts.^14^, ^15^, ^16^ Fibroblast‐like cells, derived from ECs via EndMT, play a significant part in the pathogenesis of cardiac fibrosis.^17^, ^18^ EndMT is characterized by decreased intercellular adhesion accompanied with alteration in cell polarity 19, 20 wherein endothelial markers, for example, vascular endothelial cadherin (VE‐cadherin) and CD31 are significantly down‐regulated, while mesenchymal markers, for instance, fibroblast‐specific protein‐1 (FSP‐1) and α‐smooth muscle actin (α‐SMA) are remarkably up‐regulated.^21^ SMADs (R‐SMADs, Co‐SMADs and I‐SMADs), main signal transducers of TGF‐β superfamily receptors, compose a protein family with homologous structure.^22^ SMAD2 and SMAD3 serve as R‐SMADs whereas SMAD7 belongs to I‐SMADs.^22^ EndMT is induced by activated TGF‐β1/SMAD, MAPK/ERK and PI3K/Akt, in concert with p38 MAPK signalling pathway in ECs, thereby further promoting the cardiac fibrosis phenotype of diabetic cardiomyopathy.^23^, ^24^ In contrast, inhibition of these pathways can prevent TGF‐β‐induced cardiac fibrosis.^25^ NF‐κB is a protein complex controls inflammation cytokine production and transcription of DNA, consisting of multiple members including RelA (p65) in mammalian cells.^26^ P65, encoded by RELA gene,^27^ is activated by fatty acids and hyperglycaemia in heart.^9^ Although increasing evidence suggests that high glucose concentration is associated with EndMT in ECs, the mechanism underlying the regulation of EndMT in T1DM is still unclear.^28^

miRNAs constitute a class of short, 20‐23‐nucleotide‐long, non‐coding RNA.^29^ Recently, the parts of miRNAs in cardiac fibrosis have been confirmed, thereby revealing a new mechanism underlying the regulation of cardiac diseases.^30^, ^31^ *miR‐21* has been comprehensively described in fibrosis because of its target relevance and important role in the modulation of the TGF‐β1 signalling pathway, related to the pathogenesis and progression of fibrosis in various organs.^32^, ^33^, ^34^ The association between *miR‐21* and pulmonary, renal and cardiac fibrosis has been confirmed 35, 36, 37; however, it is unclear whether *miR‐21* plays a part in EndMT and perivascular fibrosis in the heart in T1DM. The purpose of this research is to evaluate the role and the mode of *miR‐21* in regulating EndMT and cardiac perivascular fibrosis and to observe the influence of *miR‐21* inhibition on the heart of diabetic cardiomyopathy.

## 2 MATERIALS AND METHODS

### 2.1 Antibodies

All the antibodies were purchased from Abcam Company: anti‐collagen I (ab34710), anti‐collagen III (ab7778), anti‐fibronectin (ab2413), anti‐CD31 (ab24590), anti‐SMAD7 (ab216428), anti‐p‐p65 (ab86299), anti‐p‐SMAD2 (ab53100), anti‐p‐SMAD3 (ab52903) and anti‐α‐SMA (ab5694).

### 2.2 T1DM model mice

8‐week‐old to 12‐week‐old male C57BL/6 mice (Wuhan Centre for Disease Control and Prevention) were used in this research. Animal care and experimental procedures were implemented according to the NIH guidelines (publication No. 85‐23, revised 1985).

A mouse model of T1DM was generated via continuous intraperitoneal injection of streptozotocin (S0130; STZ, Sigma‐Aldrich Trading; 50 mg/kg/d) for 5 days.^38^ The mice were considered to have diabetes and were used if they developed hyperglycaemia (≥12 mmol/L).

### 2.3 *miR‐21* inhibitor treatments

For evaluating the action of *miR‐21*, mice were assigned to 3 groups: Control group (inhibitor NC), T1DM group (STZ + inhibitor NC) and T1DM + *miR‐21* inhibitor group (STZ + *miR‐21* inhibitor).

*miR‐21* inhibitor (200 nmol/kg, RioboBio) and inhibitor NC (200 nmol/kg, RioboBio) were at multiple sites intramuscularly administered into the left ventricular myocardium.^29^

### 2.4 Physiological studies

At 12 weeks after STZ injection, echocardiography was carried out by a technician in a double‐blind manner using Vevo2100 High‐Resolution Micro‐Ultrasound System (Visual Sonics).

### 2.5 Histological and immunohistochemical analyses

After physiological assessment, mice were euthanized, hearts dissected out, heart weight (HW), bodyweight (BW) and tibial length (TL) measured, followed by calculation HW/BW and HW/TL. Masson's trichrome or Sirius red staining was executed in accordance with a previously described protocol.^29^ Quantitative assessments were implemented in randomly chosen areas (200×).

For immunohistochemical analysis, paraffin‐embedded sections of mice cardiac tissues were treated with high‐pressure antigen retrieval in citrate buffer (PH = 6.0). Sections were blocked in 5% BSA then incubated with primary antibodies at a dilution ratio of 1:200: anti‐ collagen I, anti‐fibronectin, anti‐collagen III, anti‐SMAD7 and anti‐p‐p65, thereafter, incubated with corresponding HRP‐conjugated secondary antibodies (1:200, BL001A/BL003A; Biosharp). At last, nuclei were stained with haematoxylin. Pannoramic MIDIImage (3D HISTECH) was used to detect images of slides and Pro Plus6.0 for quantitative assessments.

### 2.6 In vitro analysis with HG and *miR‐21* inhibitor

Human umbilical vein endothelial cells (HUVECs; ATCC) were cultured with CC‐3162 EGM‐2 BulletKit (Lonza). Cells cultured in a 12‐well plate for 24 hours were treated with siRNA targeting p65 (RioboBio) or *miR‐21* inhibitor or cotransfected with *miR‐21* inhibitor and siRNA targeting SMAD7 (RioboBio) according to the instructions of lipofectamine 2000 (Invitrogen) and Opti‐MEM reduced serum medium (Gibco Life Technologies). 6 hours later, Opti‐MEM reduced serum medium was replaced by complete cell culture medium with high concentrations of glucose (HG: 25 mmol/L D‐glucose, G5500, Sigma, Irvine, UK) and Negative Control (NC: 25 mmol/L L‐glucose, G8644, Sigma, St. Louis, USA) for 48 hours. For further verifying the regulatory effect of p65 on *miR‐21*, HUVECs were stimulated with 20 μmol/L p65 inhibitor Quinacrine (QC) for 48 hours.

### 2.7 Immunocytochemistry and immunofluorescence staining

Mouse cardiac tissues and HUVECs were fixed with 4% paraformaldehyde. HUVECs were treated with 0.5% Triton X‐100 while cardiac tissues processed as described above, then incubated with primary anti‐α‐SMA antibody (1:200) and anti‐CD31 at 4°C after blocking with 5% BSA. The following day, cells were incubated with corresponding fluorescent antibodies: Alexa Fluor 488‐labelled Goat Anti‐Rabbit IgG (H + L) (A0423; Beyotime) and Cy3‐conjugated Goat Anti‐Mouse IgG (BA1031; Boster Biological Technology Co Ltd). Finally, nuclei were stained with DAPI. Confocal microscope was used to visualize cells.

### 2.8 Luciferase reporter assay

For assessing the activation of *miR‐21* by p65 or the suppression of SMAD7 by *miR‐21*, the binding sites were mutated using the method of site directed mutagenesis. The regions containing the predicted binding sites were amplified from human genomic DNA, cut with corresponding enzymes then subcloned into pGL3‐Basic/PMIR‐Report vectors (Applied Biosystems). Subsequently, binding sites were deleted or mutated to further validate that p65 or *miR‐21* play roles by targeting the binding site. Thereafter, p65‐p3xFLAG‐CMV‐10 and *miR‐21‐*pGL3‐Basic/*miR‐21* and SMAD7‐PMIR‐Report were cotransfected into HEK293 (ATCC) with the indicated wild‐type or mutant luciferase reporter, while Renilla acted as a transfection efficiency control.

### 2.9 qRT‐PCR

HUVEC cells cultured in 12‐well plate were transfected with mimic/mimic NC (RioboBio), inhibitor/inhibitor NC (RioboBio) or treated with TGF‐β1 at 10 ng/mL (Sigma)/TGF‐β1+ inhibitor. 48 hours later, cells were lysed with RNAiso plus (TaKaRa), while cardiac tissues were lysed with RNAiso plus and homogenized. Total RNA was reverse‐transcribed into cDNA using M‐MLV reverse transcription kit (Vazyme). qRT‐PCR was conducted with AceQ qPCR SYBR Green Master Mix (Q141‐02/03, Vazyme) on the ABI StepOnePlus™ Real‐Time PCR System. The primers sequence is as follows: *SMAD7*, 5′‐GGACGCTGTTGGTACACAAG‐3′, 5′‐GCTGCATAAACTCGTGGTCATTG‐3′; α‐SMA, 5′‐CAGGGGGCACCACTATGTAC‐3′, 5′‐CGGCTTCATCGTATTCCTGTT‐3′; CD31, 5′‐CGTGGTCAACATAACAGAACTA‐3′, 5′‐GTCCGACTTTGAGGCTATCT‐3′; *β‐actin*, 5′‐GGACTTCGAGCAGGAGATGG‐3′, 5′‐GCACCGTGTTGGCGTAGAGG‐3′.

For reverse transcription and qRT‐PCR analysis of *miR‐21*, two different Bulge‐Loop™ miRNA qRT‐PCR Primer sets were used to detect the transcriptional level of *miR‐21*, Bulge‐Loop™ *miR‐21* qPCR Primer Set and Bulge‐Loopies™ *U6* qPCR Primer Set (RioboBio).

### 2.10 Western blotting

Denatured cell lysates and myocardium samples were subjected to SDS‐PAGE. After blocking in 5% BSA, membranes were incubated with primary antibodies: anti‐fibronectin (1:3000), anti‐collagen I (1:5000), anti‐CD31 (1:1000), anti‐p‐SMAD2 (1:1000), anti‐collagen III (1:5000), anti‐p‐SMAD3 (1:3000), anti‐SMAD7 (1:2000) and anti‐α‐SMA (1:5000). After washing, membranes were incubated with corresponding secondary antibodies (BL001A/BL003A; Biosharp). The results were detected using ECL Plus and imaged with Quantity One (Bio‐Rad).

### 2.11 Statistical analyses

All the data are exhibited as mean ± SD. SPSS 19.0 (IBM) served to statistical analyses. One‐way ANOVA and Student's two‐tailed *t* test were used for multiple‐group comparisons and between‐group comparisons, respectively. The difference was statistically significant when *P* < .05.

## 3 RESULTS

### 3.1 LVEF decreased while *miR‐21* increased in myocardium of T1DM mice

Fasting glucose of T1DM mice increased significantly. Echocardiography was carried out for assessing cardiac function at 12 weeks after STZ injection. LVEF decreased significantly in T1DM mice (Figure S1A,B).

For evaluating the relationship between *miR‐21* and cardiac dysfunction, the mRNA level of *miR‐21* was assessed. *miR‐21* was up‐regulated by 2.24‐fold (*P* < .01) (Figure S1C) in the myocardium of T1DM mice.

### 3.2 Inhibiting *miR‐21* ameliorated cardiac function in T1DM mice

**Figure 1:**
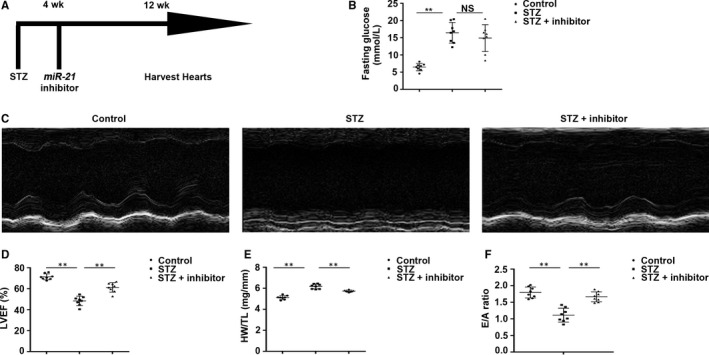
Inhibiting *miR‐21* ameliorated cardiac function of T1DM mice. A, The map of the exhibition of the assay method. B, The measurement of fasting glucose (mmol/L). **P < .01 vs Control, NS vs STZ (NS, no significant difference). C, Typical echocardiograms of two‐dimensional echocardiography, M‐mode. D, The measurement of LVEF. E, The ratio of HW/TL (mg/mm). F, The ratio of E/A. **P < .01 vs Control, **P < .01 vs STZ. n = 8 per group

Whether inhibition of *miR‐21* repressed cardiac injury in T1DM mice was investigated upon intramuscular administration of *miR‐21* inhibitor in the myocardium at 4 weeks after STZ administration. 12 weeks later, we detected the basic parameters of the three different groups including HW (mg), BW (g) and fasting glucose (mmol/L) and calculated the ratio of HW/BW (mg/g) (data not displayed) and HW/TL (mg/mm). BW (data not displayed) and fasting blood glucose of T1DM mice showed no difference whether *miR‐21* inhibitor was injected or not (Figure 1A,B). LVEF deceased in T1DM mice, while *miR‐21* inhibitor treatment rescued STZ‐induced cardiac injury (Figure 1C,D). Similar changes were observed in HW/TL (Figure 1E) and the ratio of E/A (Figure 1F).

### 3.3 Inhibition of *miR‐21* repressed cardiac mesenchymal fibrosis and perivascular fibrosis via suppressing EndMT in T1DM mice

**Figure 2:**
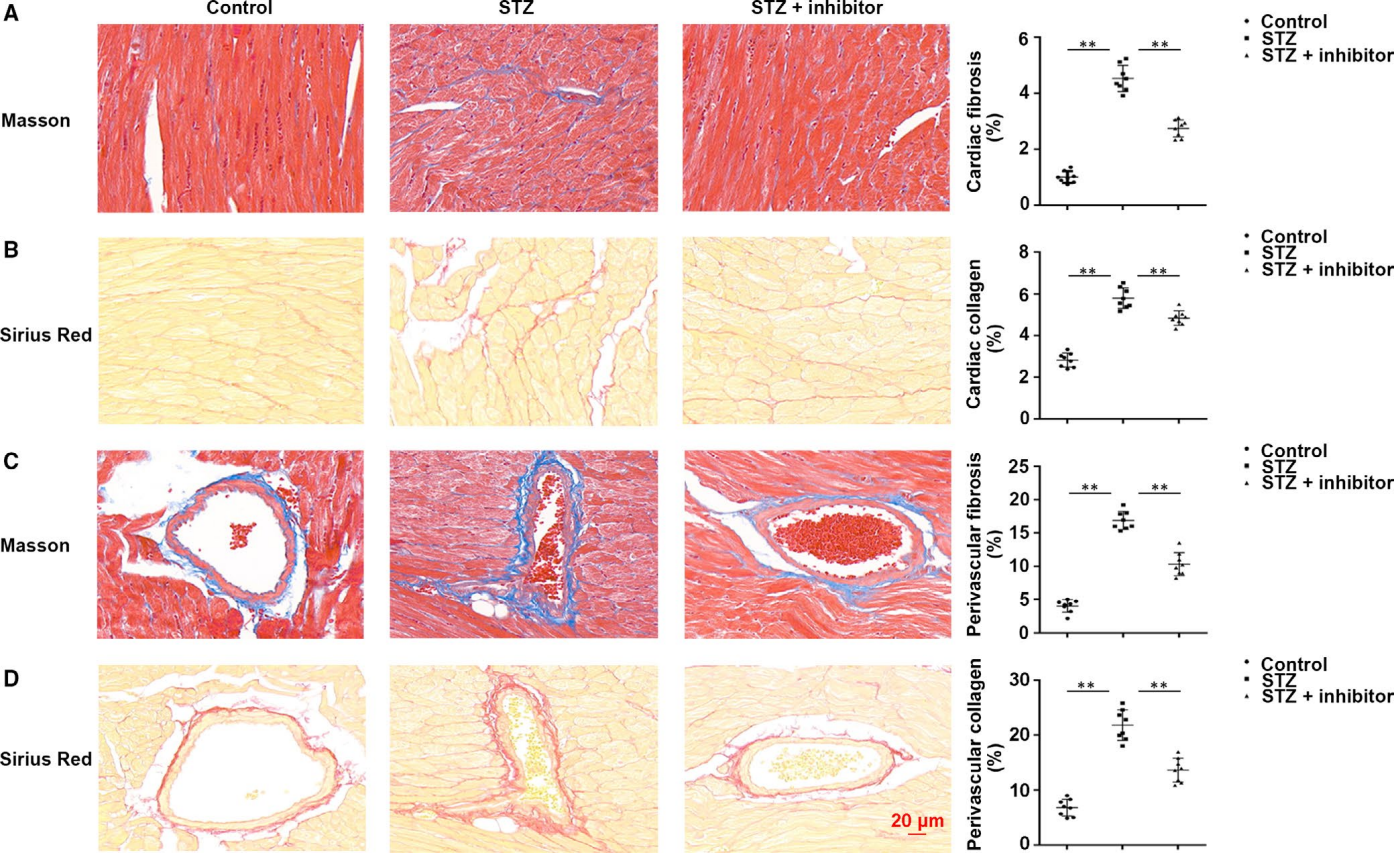
In T1DM mice, inhibiting *miR‐21* repressed STZ‐induced myocardial mesenchymal fibrosis and perivascular fibrosis. A, Masson staining of myocardial mesenchyme and the percentage of mesenchymal fibrosis. B, Sirius red staining of myocardial mesenchyme and the percentage of mesenchymal collagen. C, Masson staining of myocardial peripheral vessel and the percentage of perivascular fibrosis. D, Sirius red staining of myocardial peripheral vessel and the percentage of perivascular collagen. **P < .01 vs Control, **P < .01 vs STZ. n = 8 per group

Masson's and Sirius red staining revealed that production of the extracellular matrix was significantly increased in the myocardium in T1DM mice. Cardiac fibrosis and collagen deposition were raised by 3.83‐fold (*P* < .01) and 2.58‐fold (*P* < .01) in T1DM mice. After *miR‐21* inhibition, cardiac fibrosis and collagen deposition were decreased by 0.67‐fold (*P* < .01) and 0.76‐fold (*P* < .01). (Figure 2A,B).

Cardiac perivascular fibrosis also plays a pivotal part in cardiac dysfunction caused by hyperglycaemia. Therefore, the effects of *miR‐21* inhibitor on cardiac perivascular fibrosis were evaluated. Masson's trichrome and Sirius red staining of cardiac peripheral vessels demonstrated that ECM production was increased in the hearts of T1DM mice. Perivascular fibrosis (4.14‐fold, *P* < .01) and collagen deposition (2.86‐fold, *P* < .01) were enhanced remarkably in T1DM mice. However, *miR‐21* inhibition reduced perivascular fibrosis and collagen deposition by 0.64‐fold (*P* < .01) and 0.61‐fold (*P* < .01) (Figure 2C,D).

**Figure 3:**
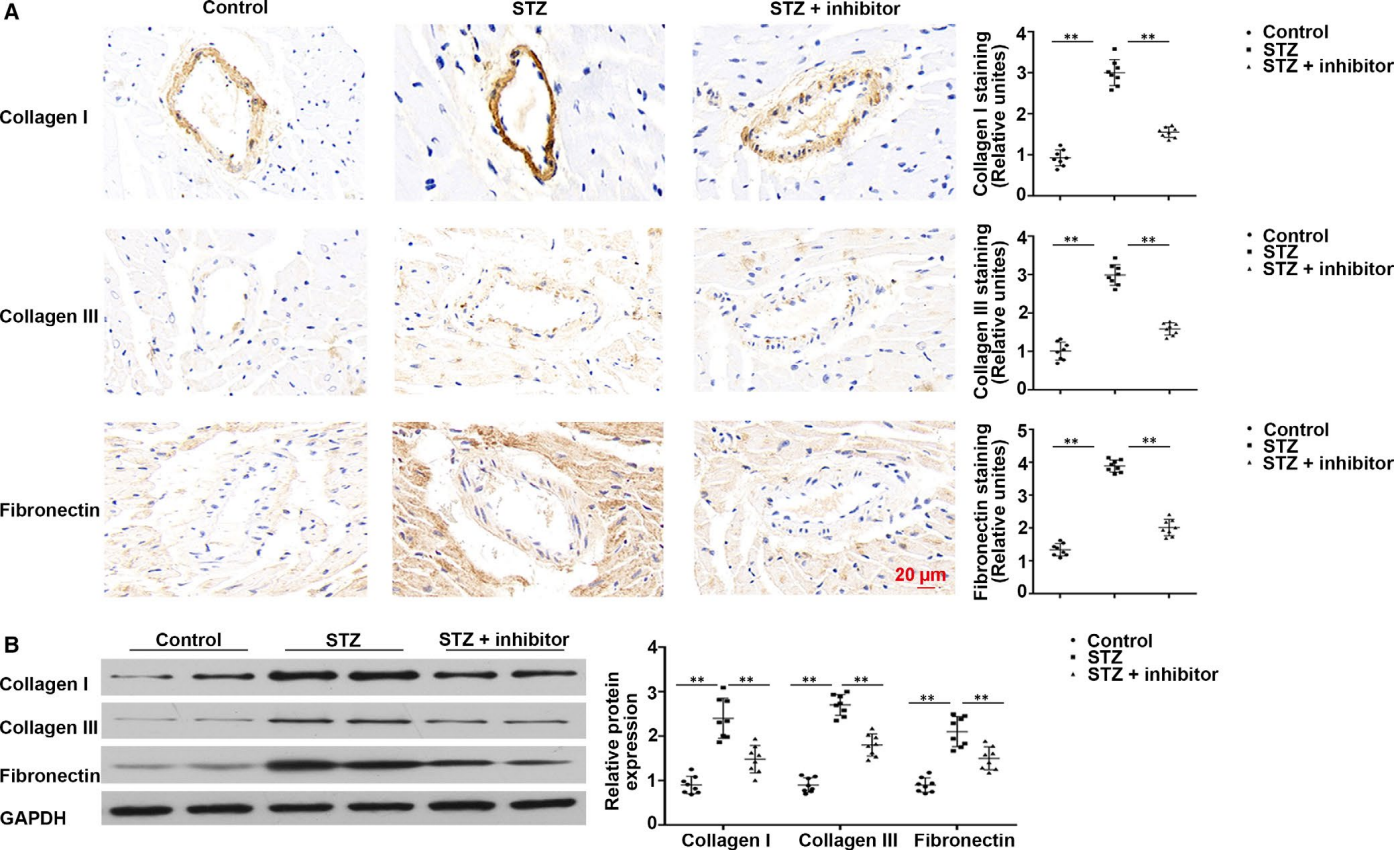
Fibrotic markers were increased in T1DM mice. A, Immunohistochemical staining of collagen I, collagen III and fibronectin. B, Western blotting of collagen I, collagen III and fibronectin in cardiac tissues. **P < .01 vs Control, **P < .01 vs STZ. n = 8 per group

Diabetes mellitus up‐regulated fibrotic markers collagen I, collagen III in addition to fibronectin, but *miR‐21* inhibitor reduced cardiac fibrosis (Figure 3A). Furthermore, Western blotting revealed that collagen I (*P* < .01), collagen III (*P* < .01) and fibronectin (*P* < .01) were prominently up‐regulated in T1DM mice while markedly down‐regulated upon *miR‐21* inhibition (collagen I, *P* < .01; collagen III, *P* < .01; fibronectin, *P* < .01) (Figure 3B).

**Figure 4:**
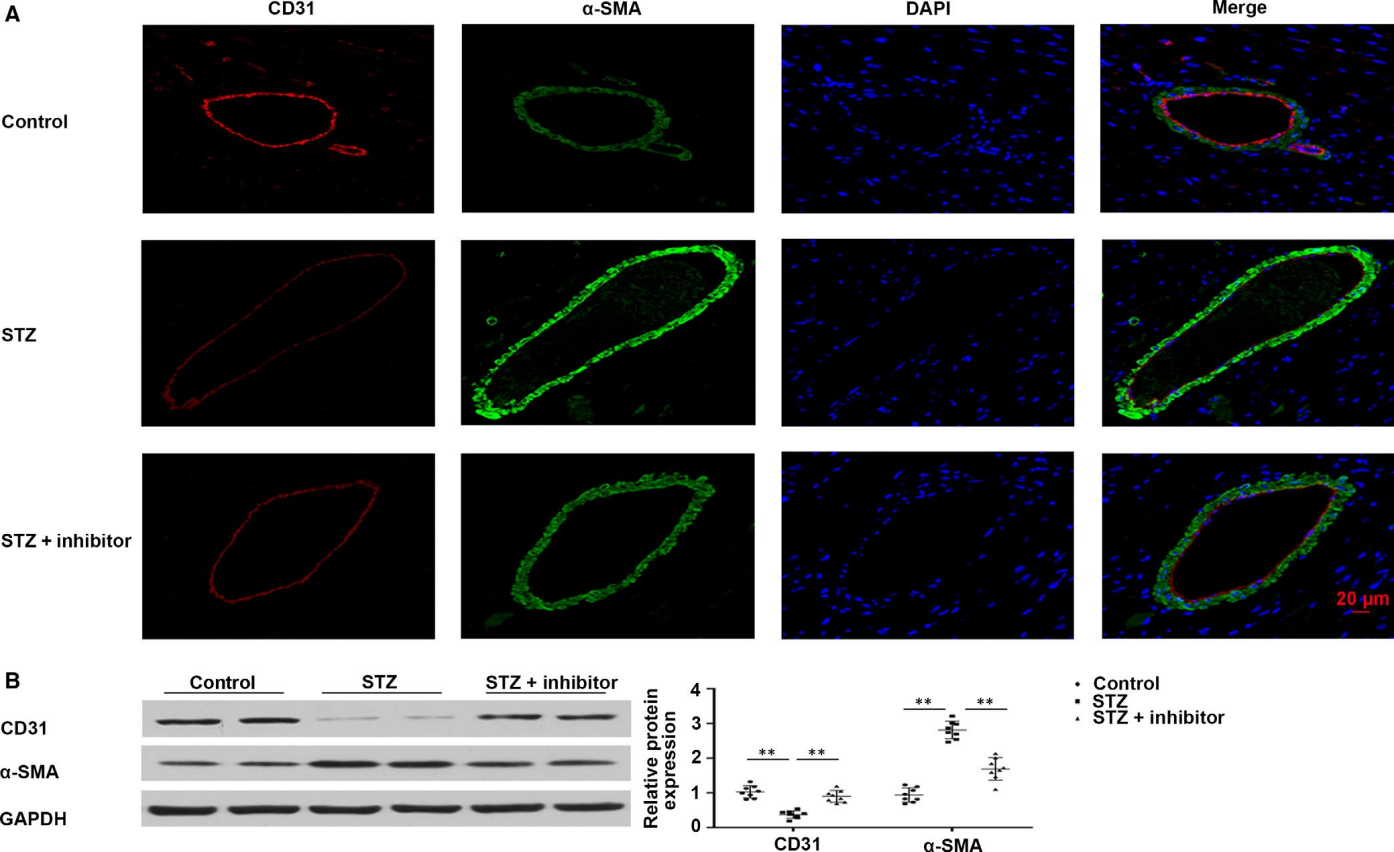
*miR‐21* inhibition alleviated the fibrosis of myocardial peripheral vessels in T1DM mice. A, Immunofluorescence co‐staining of CD31 and α‐SMA in cardiac tissues. B, Western blotting of CD31 and α‐SMA in myocardial tissues. **P < .01 vs Control, **P < .01 vs STZ. n = 8 per group

Endothelial cells lost their features during EndMT, for instance, CD31 expression, but acquired mesenchymal features, for example, α‐SMA expression. For estimating the action of *miR‐21* inhibition on EndMT in the myocardium of T1DM mice, immunofluorescence co‐staining of CD31 and α‐SMA and western blotting assays were performed. We found that CD31 was markedly down‐regulated in myocardial vessels in T1DM mice; this down‐regulation was reversed upon administration of *miR‐21* inhibitor. In contrast, mesenchymal marker α‐SMA was up‐regulated in cardiac tissues of T1DM mice; however, this up‐regulation was reversed upon *miR‐21* inhibition (Figure 4A,B). These results suggest that *miR‐21* inhibition may partially alleviate EndMT in the heart of T1DM mice.

### 3.4 Inhibiting *miR‐21* attenuated the STZ‐induced SMAD pathway in the cardiac tissues in T1DM mice

**Figure 5:**
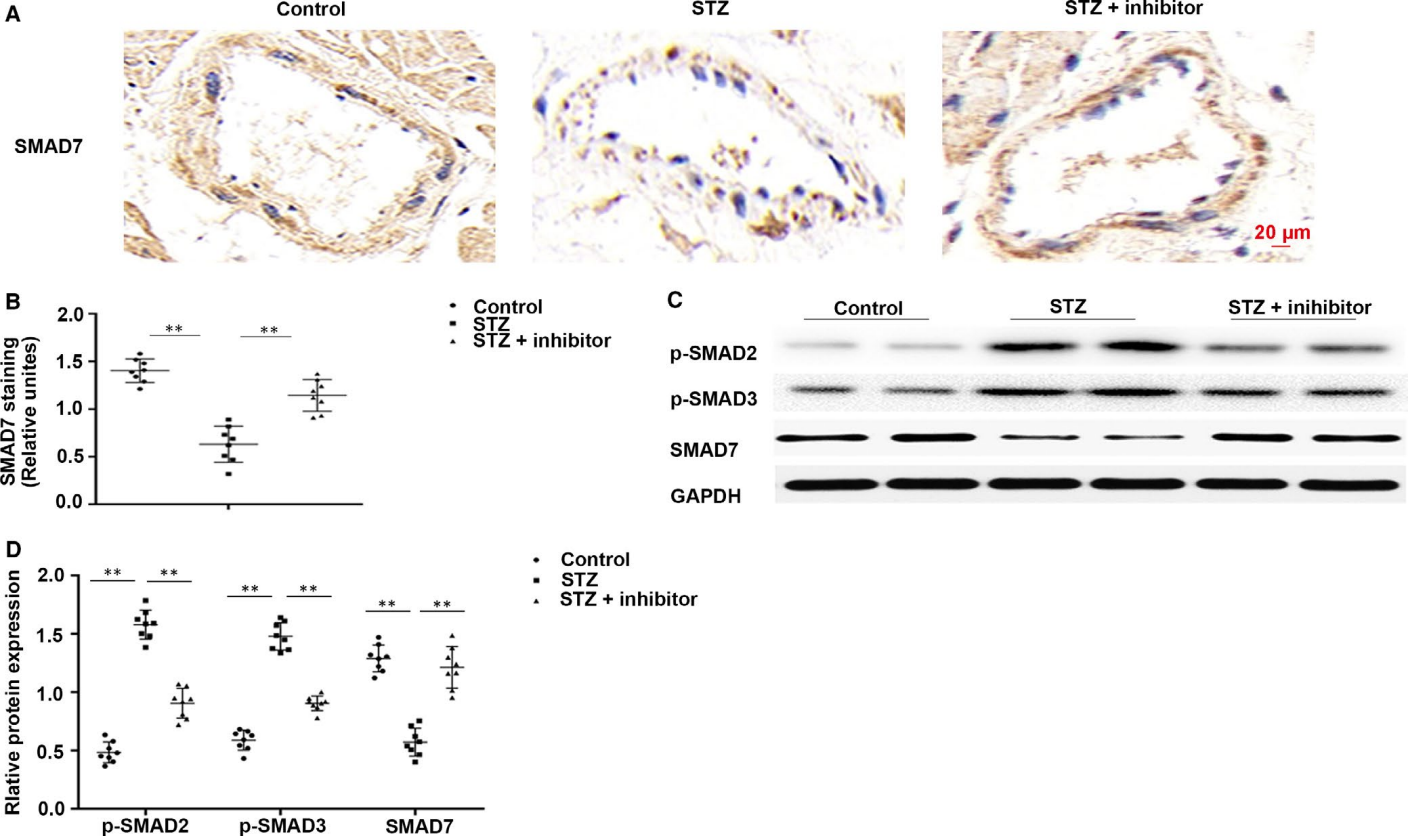
*miR‐21* inhibition attenuated the STZ‐induced SMAD pathway in the myocardial tissues in T1DM mice. (A,B) Immunohistochemical staining of SMAD7 in cardiac tissues. (C,D) Western blotting of p‐SMAD2, p‐SMAD3 and SMAD7 in cardiac tissues. **P < .01 vs Control, **P < .01 vs STZ. n = 8 per group

Previous studies have reported that *miR‐21* is of key importance in CFs activation and cardiac fibrosis after myocardial infarction by targeting SMAD7.^39^ Herein, immunohistochemical analysis revealed that SMAD7 was significantly down‐regulated in T1DM mice, while inhibition of *miR‐21* up‐regulated SMAD7 in perivascular tissue in diabetic myocardium (Figure 5A,B). Western blotting revealed that p‐SMAD2 and p‐SMAD3 were markedly up‐regulated, while SMAD7 was observably down‐regulated in T1DM mice. However, in *miR‐21* inhibitor‐treated mice, the activation of p‐SMAD2 and p‐SMAD3 was markedly reduced, but that of SMAD7 was observably increased. The results indicated that inhibition of *miR‐21* may suppress the activation of the p‐SMAD2 and p‐SMAD3 pathway in the heart of T1DM mice via up‐regulation of SMAD7 (Figure 5C,D).

### 3.5 *miR‐21* is positively regulated by p65

Because blood glucose is elevated in patients and mice with T1DM, HUVECs treated with high glucose concentrations were used to elucidate the mechanism underlying changes in myocardial *miR‐21* expression in T1DM mice.

**Figure 6:**
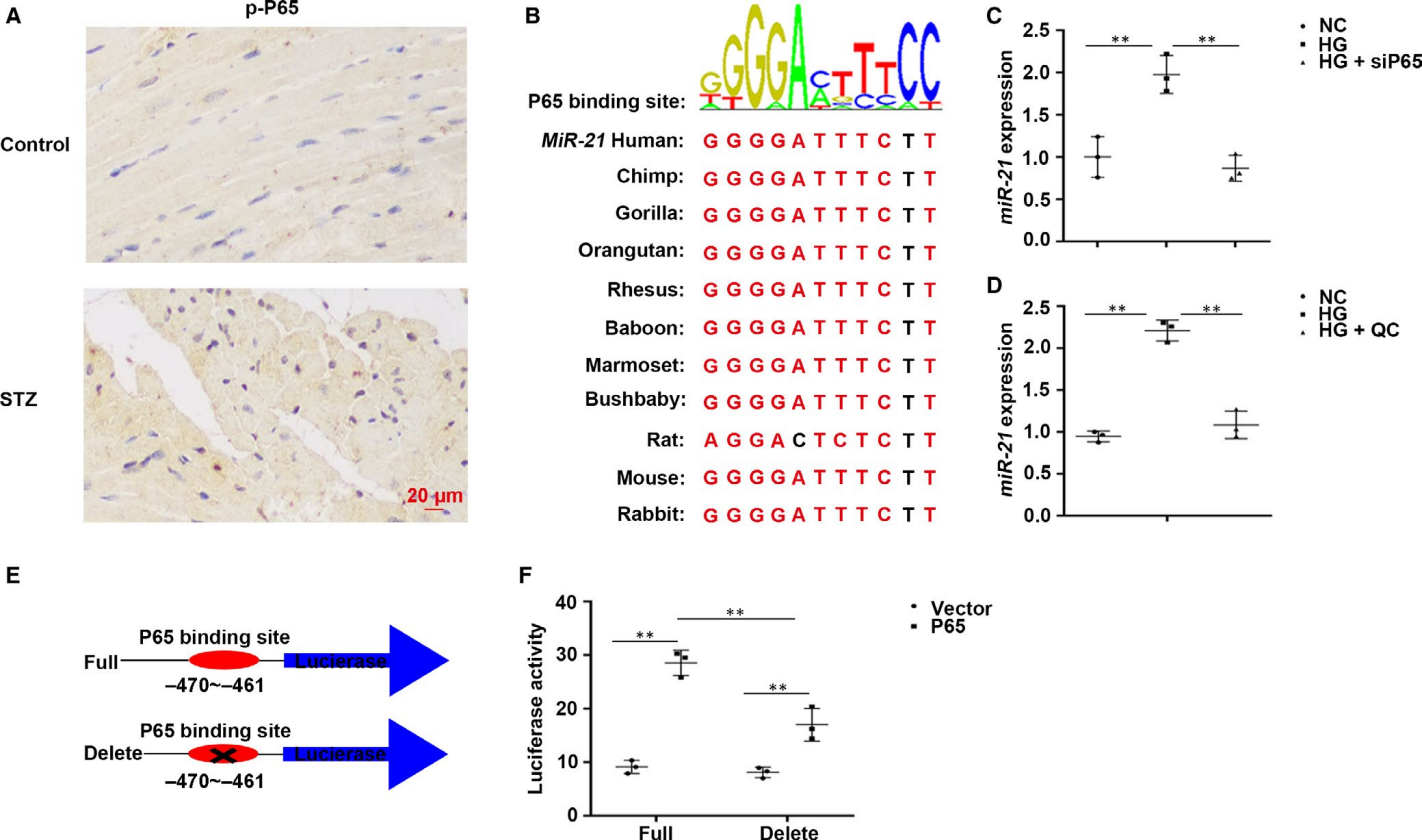
*miR‐21* is positively regulated by p65. A, Immunohistochemical staining of p‐p65 in cardiac tissues, n = 8 per group. B, The binding site of p65 and the conservatism prediction of the binding site in different species. (C,D) The expression of *miR‐21* in HUVECs. (E,F) Luciferase activity of *miR‐21* in HEK293 cells

Immunohistochemical results of p‐p65 in mouse cardiac tissues confirmed that the expression of p‐p65 was activated in T1DM mice (Figure 6A). Conservatism prediction revealed that the binding site of p65 is evolutionarily conserved across taxa (Figure 6B). The results of qRT‐PCR revealed that *miR‐21* was markedly up‐regulated upon treatment with HG and evidently down‐regulated after siRNA for p65 (Figure 6C) or after treatment with p65 inhibitor QC (Figure 6D).

TRED database was used for retrieving the sequence of the *miR‐21* promoter and regulatory region. Via PROMO 3.0 and ConTraV2, we identified that the physical position −470 to −461 at the 5′‐UTR of *miR‐21* was the binding site for p65 (Figure 6E). The luciferase activity of *miR‐21* was significantly increased under the action of p65, which was decreased upon deletion p65 binding site (Figure 6F).

### 3.6 SMAD7 is directly regulated by *miR‐21* in HUVECs

SMAD7 is directly regulated by *miR‐21* in CFs and mesenchymal stem cells, however, whether it is still the direct target of *miR‐21* in HUVECs remains unclear and how the pathophysiology regulation mode of perivascular fibrosis is.

Herein, TargetScan7.2 was used to identify potential target genes regulating fibrosis. SMAD7 is one of the candidate target genes related to EndMT with an 8‐nucleotide binding site in different species (Figure S2A). To determine whether *miR‐21* inhibits SMAD7 expression by binding to the 3′‐UTR, qRT‐PCR was carried out to investigate the effect of *miR‐21* on the expression of *SMAD7* in HUVECs. The results indicated that *SMAD7* mRNA levels remained largely unchanged (Figure S2B). Furthermore, the luciferase activation of WT was significantly repressed by *miR*‐21 compared with the mutant SMAD7 (Figure S2C,D). Western blotting revealed that SMAD7 was significantly down‐regulated when treated with *miR‐21* (Figure S2E,F), while notably up‐regulated when treated with *miR‐21* inhibitor in HUVECs (Figure S2E,F). All results suggested that SMAD7 is directly regulated by *miR‐21* in HUVECs.

### 3.7 *miR‐21* inhibition repressed HG‐mediated EndMT in vitro via regulation of SMAD pathway

**Figure 7:**
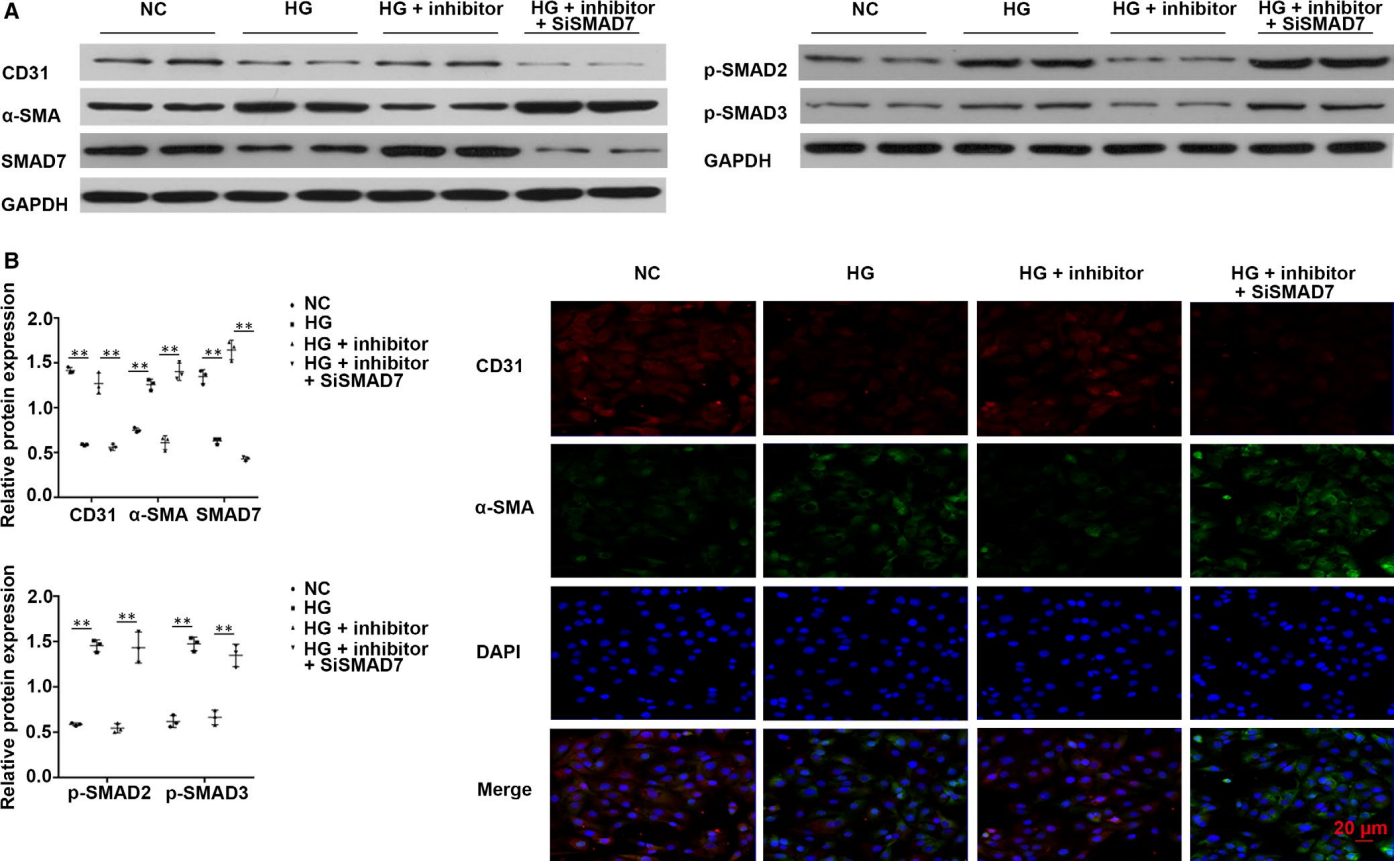
*miR‐21* inhibitor suppressed HG‐induced EndMT via regulating SMAD pathway. (A,B) Western blotting of CD31, α‐SMA, SMAD7, p‐SMAD2 and p‐SMAD3 in HUVECs. C, Immunofluorescent staining of CD31 and α‐SMA in HUVECs

Western blotting revealed that CD31 and SMAD7 were markedly down‐regulated (*P* < .01) with the increase in glucose concentration; however, α‐SMA was significantly up‐regulated (*P* < .01) in HUVECs, which was confirmed by immunofluorescence of CD31 and α‐SMA. Moreover, p‐SMAD2 and p‐SMAD3 were significantly activated by HG. These events were reversed upon treatment with *miR‐21* inhibitor. After siRNA‐mediated knockdown of SMAD7, the protective effect of *miR‐21* inhibition was diminished (Figure 7A‐C). *miR‐21* inhibition also prevented EndMT caused by TGF‐β1 (10 ng/mL) (Figure S3). These results indicated that *miR‐21* inhibitor may inhibit EndMT in vitro.

## 4 DISCUSSION

Previous reports have confirmed that *miR‐21* is closely related to organ fibrosis induced by diabetes mellitus. Clinic and animal studies revealed a significant increase in *miR‐21* in plasma and urine of patients with T1DM, and up‐regulation of plasma *miR‐21* levels in young diabetic patients may be an indicator of fibrosis remodelling that already exists.^40^ It is well known that *miR‐21* can induce fibrosis in many organs, for instance, kidney and heart 41, 42 and promote collagen synthesis in fibroblasts treated with HG.^43^

We observed that increased *miR‐21* may induce both cardiac mesenchymal fibrosis and cardiac perivascular fibrosis through SMAD7 signalling pathway and inhibition of *miR‐21* decreased cardiac fibrosis induced by hyperglycaemia and prevented cardiac structural and functional abnormalities in T1DM mice. Furthermore, we identified a highly conserved binding site of NF‐κB in *miR‐21* 5′‐UTR and demonstrated that HG up‐regulated *miR‐21* in HUVECs via up‐regulation of p65.

The action of EndMT in diabetic myocardial fibrosis has been confirmed in both T1DM and T2DM mice.^14^ Endothelial injury resulting from hyperglycaemia causes phenotypic changes in EndMT, playing a vital part in the process of myocardial fibrosis 44 EndMT participates in cell initiation of myofibroblasts and in ECM proteins secretion during the occurrence and development of myocardial fibrosis, which is a unique feature of dilated cardiomyopathy.^20^

In the present study, endothelial marker was down‐regulated and fibroblast marker up‐regulated, and these changes are correlated with *miR‐21* up‐regulation.

*miR‐21* has a wide range of functions. By targeting PDCD4, *miR‐21* participated in preventing cardiomyocyte death.^45^ FasL or PTEN belonging to anti‐apoptotic gene is also the target of *miR‐21*.^46^ *miR‐21* participates in the process of angiogenesis and repair of inflammation or ischaemic injury by reducing NF‐κB or through PTEN/AKT/ERK1‐VEGF pathway.^46^ Excessive expression of *miR‐21* in bone marrow‐derived mesenchymal stem cells can effectively repair myocardial injury in rats.^47^ In the upstream, *miR‐21* was regulated by HIF1A,^48^ binding together with PER2, which is vital for regulating HIF1A target genes, in hypoxia.^49^

SMAD7 is directly regulated by *miR‐21* in CFs.^39^ A population genetic research reported that *SMAD7* may be associated with the pathogenesis of T1DM and T2DM. In T2DM, an intronic variant IVS2‐21 C > T of *SMAD7* is associated with the disease under the recessive genetic model.^50^ Merriman *et al* suggested an association between chromosome 18q12‐q21 region (*SMAD7* locates) with T1DM in 882 families,^51^ and Barrett *et al* confirmed the association between rs12953717, a tag SNP (*P* = 10^−6^) for *SMAD7*, and T1DM in a genome‐wide association study among Caucasians.^52^ Furthermore, a study involving 928 T1DM patients and 922 control patients revealed that three genes, including *SMAD7*, were down‐regulated in T1DM patients.^53^

In HUVECs, SMAD7 was confirmed remains a direct target of *miR‐21*. SMAD7 was down‐regulated; however, *miR‐21* up‐regulated significantly in the myocardium of T1DM mice, while these changes were reversed after inhibiting *miR‐21*. Together, inhibition of *miR‐21* may up‐regulate SMAD7 and improve both cardiac mesenchymal fibrosis and cardiac perivascular fibrosis, thereby promoting myocardial function in T1DM.

SMAD7 attenuates TGF‐β/SMAD signal transduction, which is reported to be a classic pathway to participate in the activation of EndMT.^54^ The specific receptors (TGF‐βRI/TGF‐βRII) of TGF‐β are activated by TGF‐β, subsequently stimulating phosphorylation of SMAD2 and SMAD3.^55^ Complexes formed by p‐SMAD2, p‐SMAD3 and SMAD4 translocate from cytoplasm to nucleus regulating downstream targets containing SMAD7.^56^

Herein, SMAD7 was down‐regulated, whereas the phosphorylation of SMAD2 and SMAD3 enhanced in the myocardium in T1DM mice and in HUVECs. In addition, endothelial marker was down‐regulated and fibroblast marker up‐regulated, suggesting that hyperglycaemia‐induced EndMT may play a part of the function in cardiac perivascular fibrosis in T1DM. Inhibition of *miR‐21* significantly decreased high glucose‐induced EndMT in HUVECs, while SMAD7 knockdown blunted the effect of *miR‐21* inhibitor. These results suggested that inhibition of *miR‐21* may reverse hyperglycaemia‐induced cardiac perivascular fibrosis via regulation of SMAD7.

In the present study, a model was proposed to interpret the action of *miR‐21* in perivascular fibrosis caused by hyperglycaemia (Figure S4). Overall, hyperglycaemia up‐regulated *miR‐21*, followed by down‐regulation of its target SMAD7, which, in turn, enhanced the phosphorylation of SMAD2 and SMAD3, thereby promoting EndMT activation and myocardial fibrosis. To our knowledge, this is the first study to reveal the part of *miR‐21* in regulating myocardial perivascular fibrosis in T1DM.

Our study has the following limitations. Although the results suggest that *miR‐21* plays an important regulatory part in cardiac perivascular fibrosis, the relationship between *miR‐21* and SMAD7 did not display one‐to‐one stoichiometry. Furthermore, miRNAs have numerous target genes, and our study has not ruled out the possibility that *miR‐21* promotes the pathogenesis of cardiac perivascular fibrosis by simultaneously targeting other genes in T1DM; the present results merely confirm that SMAD7 brings into play in this process. In the future research, the role of *miR‐21* and other target genes besides SMAD7 in the EndMT process and the specific molecular mechanism will be focused on. More importantly, our existing research lacks an endothelial cell (EC)‐specific *miR‐21* knockout mice experiments, since *miR‐21* inhibitor is known to have little or no specificity for cell types when administered to mice in vivo. Therefore, further experimental studies are needed to address this issue. Although population‐level evidence confirms that both *miR‐21* and SMAD7 are closely associated with T1DM and T2DM, whether SMAD7 expression is affected by *miR‐21* in the long‐term, and the exact role of *miR‐21* and SMAD7 in myocardial fibrosis and heart failure in patients with T1DM needs more evidence in the future prospective clinic studies. Since STZ‐induced diabetic mice exhibited changes in cardiac systolic function at 8^57^ or 12 weeks,^58^ in the present research, the changes of cardiac systolic function were detected at 12 weeks after induction of T1DM. Diabetes mellitus has chronic and long‐term cardiac damage, so the long‐term effects of *miR‐21* inhibitor on diabetic cardiac injury (24 and 36 weeks) need to be evaluated in future.

This study indicated that inhibiting *miR‐21* may prevent myocardial perivascular fibrosis and ameliorate myocardial function of T1DM partially by inhibiting EndMT. Potential mechanism may involve NF‐κB/*miR‐21*/SMAD7 signalling pathway. The precise potential mechanism is yet unclear, but inhibition of *miR‐21* may be a lurking therapeutic target for diabetic cardiac complications and multiple organ damage. Furthermore, EndMT contributes to fibrosis pathogenesis in different organs, inhibition of *miR‐21* may be a new therapeutic target for multiple organ damage in diabetes mellitus.

## CONFLICT OF INTEREST

The authors confirm that there are no conflicts of interest.

## AUTHOR CONTRIBUTIONS

Q‐QL, Y‐FY and XT designed the research, carried out the data analysis, wrote the paper and assisted in preparation of the manuscript; Q‐QL, Y‐FY, S‐MS, M‐CZ, Y‐CZ and M‐RW carried out the experimental work;; JC, Z‐RH, W‐LZ, ML, and QW revised the manuscript critically.

## Data Availability

The data supporting the findings of this study are available within the article and its supplementary information files.
