# Supplementary material for: Inhibition of miR‐21 alleviated cardiac perivascular fibrosis via repressing EndMT in T1DM
Source: J Cell Mol Med. 2019 Nov 3;24(1):910–20. doi: 10.1111/jcmm.14800 (PMC6933373; doi:10.1111/jcmm.14800)
Supplement: Supplementary file 1 [file JCMM-24-910-s001.doc]

**
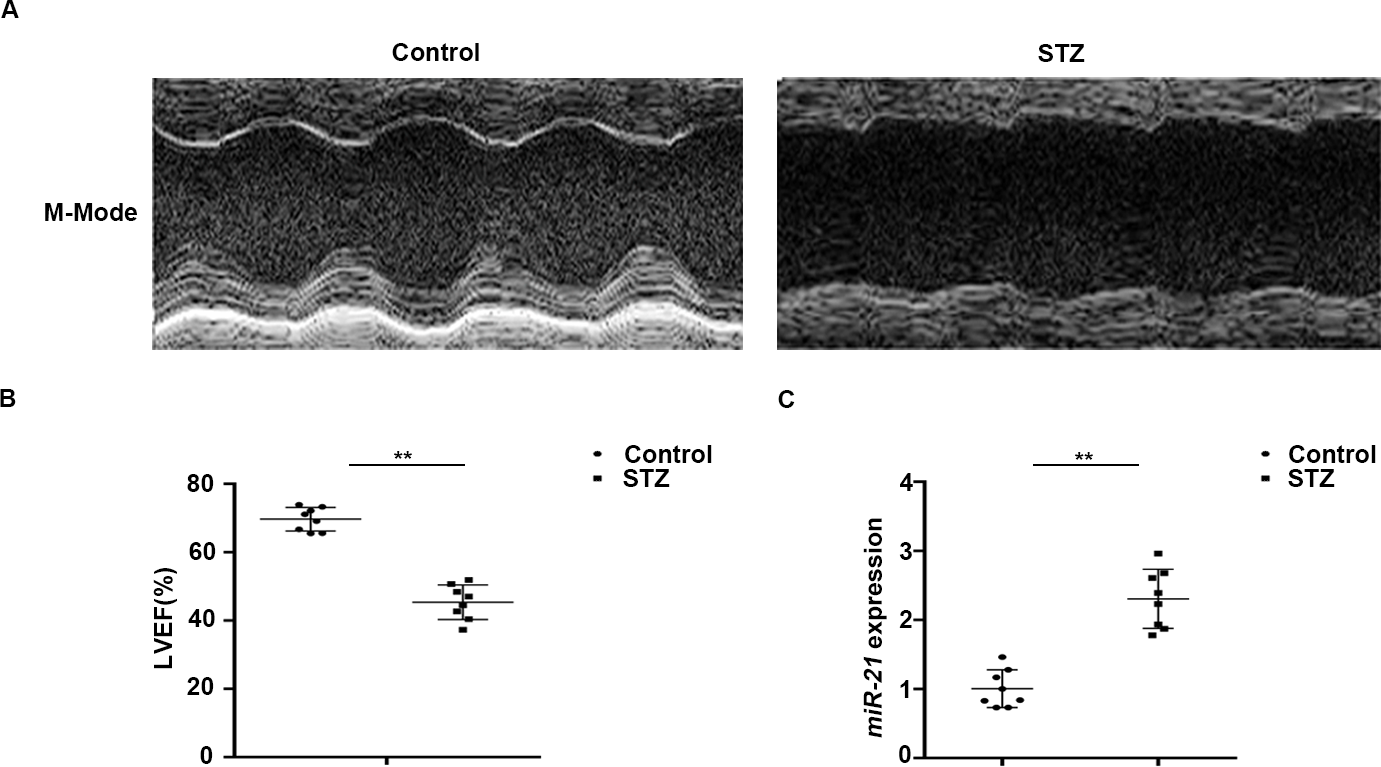
**

**Supplementary Figure 1** *MiR-21* expression was up-regulated in the hearts of T1DM mice. (A) Typical echocardiograms of two-dimensional echocardiography, M-mode. (B) The measurement of LVEF. (C) The expression of *miR-21* in the hearts of T1DM mice. ***P*<0.01 *vs.* Control. n=8 per group.

**
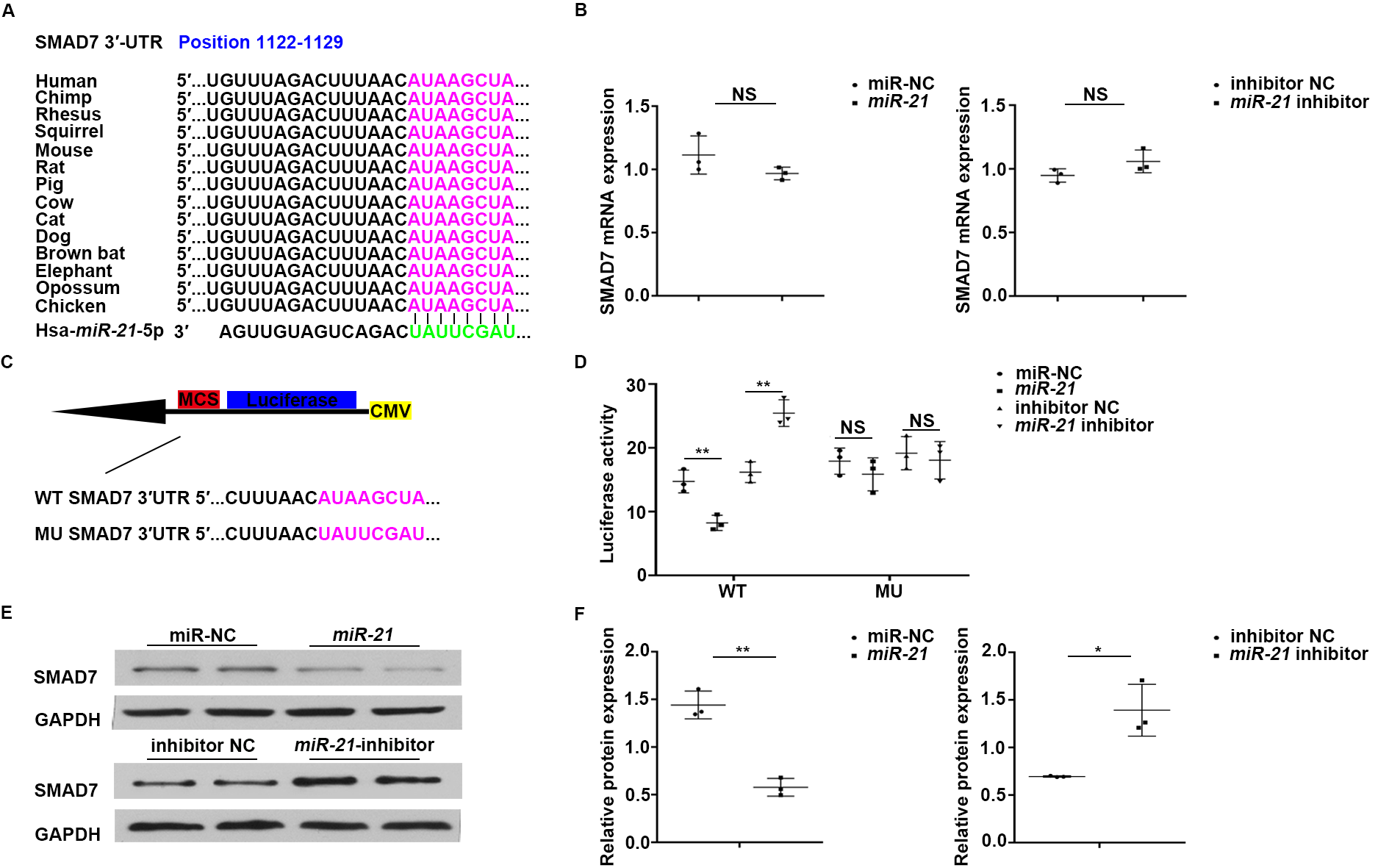
Supplementary Figure 2** SMAD7 is directly regulated by *miR-21* in HUVECs (A) The binding site of *miR-21* and the conservatism prediction in different species. (B) The results the mRNA expression of SMAD7 after transfection of *miR-21* mimic and inhibitor in HUVECs. (C) The map of the exhibition of the binding site (WT and MU). (D) Luciferase reporter assay in HEK293 cells. NS, no significant difference, ***P*<0.01 (n=3). (E), (F) Western blotting of SMAD7 after transfection of *miR-21* mimic and inhibitor in HUVECs, ***P*<0.01 *vs.* miR-NC; **P*<0.05 *vs.* inhibitor NC.

**
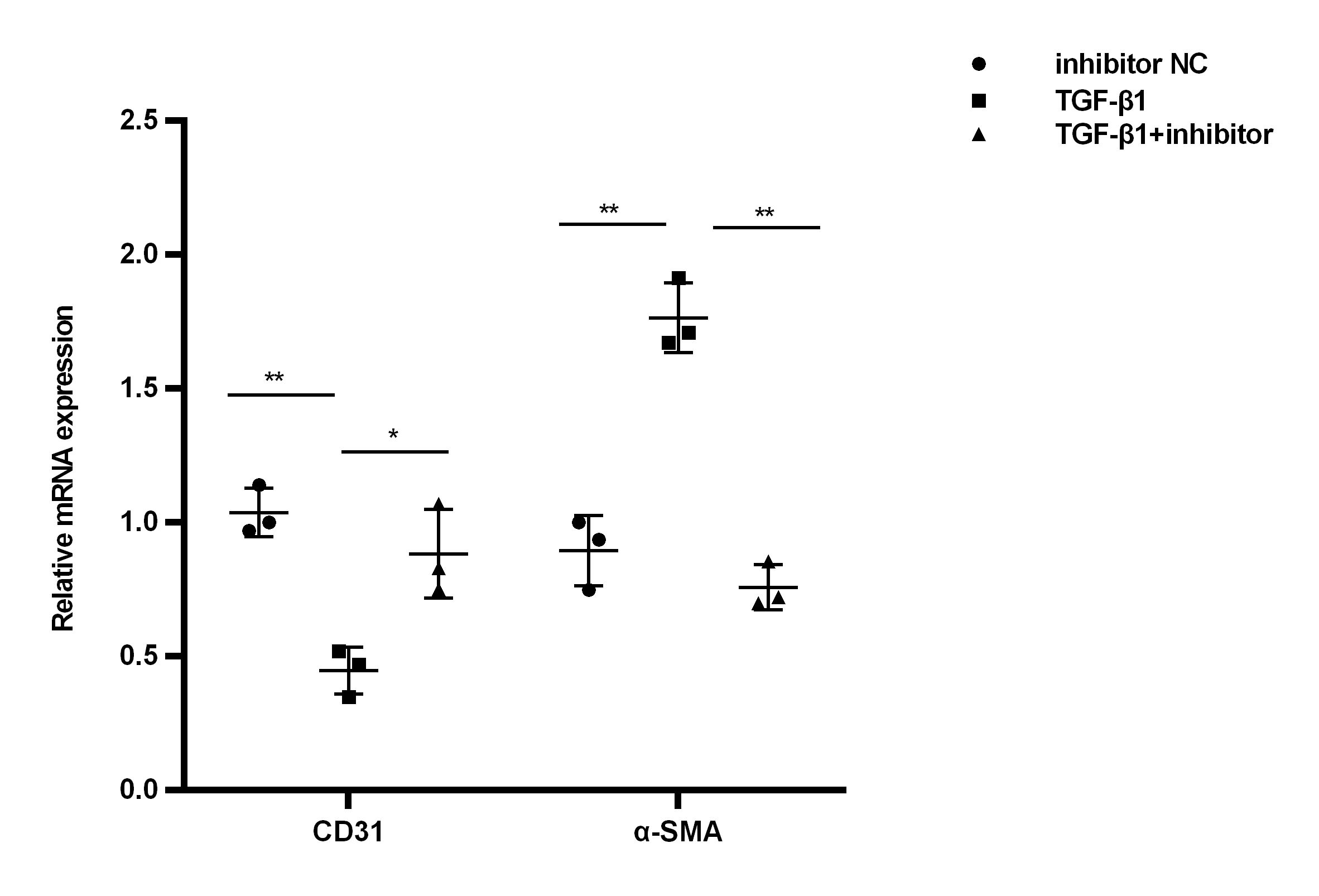
**

**Supplementary Figure 3** The results the mRNA expression of α-SMA and CD31 in HUVECs (TGF-β1 10 ng/ml).


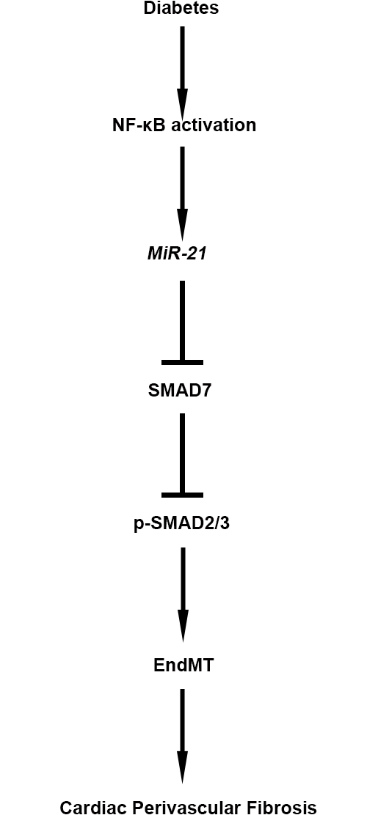


**Supplementary Figure 4** The map of the exhibition of the signal pathway of hyperglycemia-induced cardiac perivascular fibrosis.
